# Endogenous Epoxygenases Are Modulators of Monocyte/Macrophage Activity

**DOI:** 10.1371/journal.pone.0026591

**Published:** 2011-10-19

**Authors:** Jonas Bystrom, Jessica A. Wray, Mary C. Sugden, Mark J. Holness, Karen E. Swales, Timothy D. Warner, Matthew L. Edin, Darryl C. Zeldin, Derek W. Gilroy, David Bishop-Bailey

**Affiliations:** 1 William Harvey Research Institute, Queen Mary University London, London, United Kingdom; 2 Blizzard Institute of Cell and Molecular Science, Barts and the London, Queen Mary University London, London, United Kingdom; 3 Division of Intramural Research, National Institute of Environmental Health Sciences/National Institutes of Health, Research Triangle Park, North Carolina, United States of America; 4 Division of Medicine, University College London, London, United Kingdom; Fundação Oswaldo Cruz, Brazil

## Abstract

**Background:**

Arachidonic acid is metabolized through three major metabolic pathways, the cyclooxygenase, lipoxygenase and CYP450 enzyme systems. Unlike cyclooxygenase and lipoxygenases, the role of CYP450 epoxygenases in monocyte/macrophage-mediated responses is not known.

**Methodology/Principal Findings:**

When transfected in vitro, CYP2J2 is an efficient activator of anti-inflammatory pathways through the nuclear receptor peroxisome proliferator-activated receptor (PPAR) α. Human monocytes and macrophages contain PPARα and here we show they express the epoxygenases CYP2J2 and CYP2C8. Inhibition of constitutive monocyte epoxygenases using the epoxygenase inhibitor SKF525A induces cyclooxygenase (COX)-2 expression and activity, and the release of TNFα, and can be reversed by either add back of the endogenous epoxygenase products and PPARα ligand 11,12- epoxyeicosatrienoic acid (EET) or the addition of the selective synthetic PPARα ligand GW7647. In alternatively activated (IL-4-treated) monocytes, in contrast to classically activated cells, epoxygenase inhibition decreased TNFα release. Epoxygenases can be pro-inflammatory via superoxide anion production. The suppression of TNFα by SKF525A in the presence of IL-4 was associated with a reduction in superoxide anion generation and reproduced by the superoxide dismutase MnCl_2_. Similar to these acute activation studies, in monocyte derived macrophages, epoxygenase inhibition elevates M1 macrophage TNFα mRNA and further decreases M2 macrophage TNFα.

**Conclusions/Significance:**

In conclusion, epoxygenase activity represents an important endogenous pathway which limits monocyte activation. Moreover endogenous epoxygenases are immuno-modulators regulating monocyte/macrophage activation depending on the underlying activation state.

## Introduction

Monocyte-derived macrophages play a critical role in host defence, wound healing, repair and chronic inflammation [1]. Depending on the stimuli monocytes can be differentiated to either a classically activated pro-inflammatory M1 macrophage (e.g. by IFNγ, TNFα or bacterial LPS) or an alternatively activated M2 macrophage (M2; e.g. by IL-4 or IL-13) which in general are associated with Th2 mediated immune responses, promote the killing of parasites, and are present during tissue repair, wound healing and remodelling [1].

Arachidonic acid is metabolised in to families of biologically active mediators by cyclooxygenase (COX), lipoxygenase and CYP450 pathways [2], [3]. Unlike COX and lipoxygenase products, the roles of CYP450 pathways in the immune response are poorly understood. CYPs metabolise arachidonic acid by: i) epoxygenases that catalyze arachidonic acid to epoxyeicosatrienoic acids (EETs); ii) the lipoxygenase-like CYPs; and iii) the ω- and ω-1-hydroxylase CYPs which produce hydroxyeicosatetraenoic acids.[3] These CYP-lipid metabolizing enzymes are the primary sources of eicosanoids in small blood vessels, the kidney, liver, lung, intestines, heart, and pancreas [3], [4]. The main arachidonic acid-metabolising CYPs are members of the CYP2 family. Of this larger family, the CYP2J and CYP2C sub families represent the most important epoxygenases [3], [4], [5], [6].

EETs can regulate vascular tone, smooth muscle cell mitogenesis, platelet aggregation, steroidogenesis and vascular inflammation [4], [6], [7]. EETs have been hypothesized as endothelium-derived hyperpolarizing factors, hyperpolarizing and relaxing vascular smooth muscle cells by activating calcium-activated potassium channels [5]. A number of the anti-inflammatory activities of EETs and CYP2J2 in particular are independent of hyperpolarisation [8]. CYP2C9 increases NFκB activity in human vascular endothelium [9], giving CYP2C9 a potential pro-inflammatory profile. Indeed, in general, CYP2Cs have a propensity to uncouple and produce reactive oxygen species whereas CYP2J isoforms do not. The receptors for epoxygenase products are poorly understood. We recently published that CYP2J2 and its anti-inflammatory products are ligands for the peroxisome-proliferator activated receptor (PPAR) class of nuclear receptors, in particular PPARα [10]. PPARα is expressed in the vascular endothelium, vascular smooth muscle cells, and monocyte/macrophages where, like EETs, it can limit NFκB activation [11].

Over the last 10 years it has been recognized that endothelial cells contain epoxygenases (in man CYP2J2, CYP2C8 and CYP2C9), which regulate endothelial cell adhesion molecule expression and inflammatory cell recruitment [4]. CYP2J2 in particular has a number of vascular protective properties including protection of hypoxia-reoxygenation endothelial injury [12], decreased cytokine-induced cell adhesion molecule expression by inhibition of NFκB [8], reduction of inflammatory cell recruitment [13], and the reduction of hypertension and hypertension induced-renal injury [14] and smooth muscle migration [15]. In contrast, the expression and function of epoxygenases in monocytes is poorly understood, even though it is known epoxygenase products are produced by human macrophages [16] and bind to sites in monocytes [17]. Our aims were to examine whether epoxygenases played a immuno-modulatory role in monocyte macrophage activity. We show endogenous epoxygenase activity limits monocyte/macrophage activation when stimulated either in a classical pro-inflammatory manner or with alternative activation. Endogenous epoxygenases are therefore important immuno-modulators regulating monocyte/macrophage activation depending on the manner of their activation.

## Results

### Transfected CYP2J2 activates anti-inflammatory pathways through PPARα

**Figure 1:**
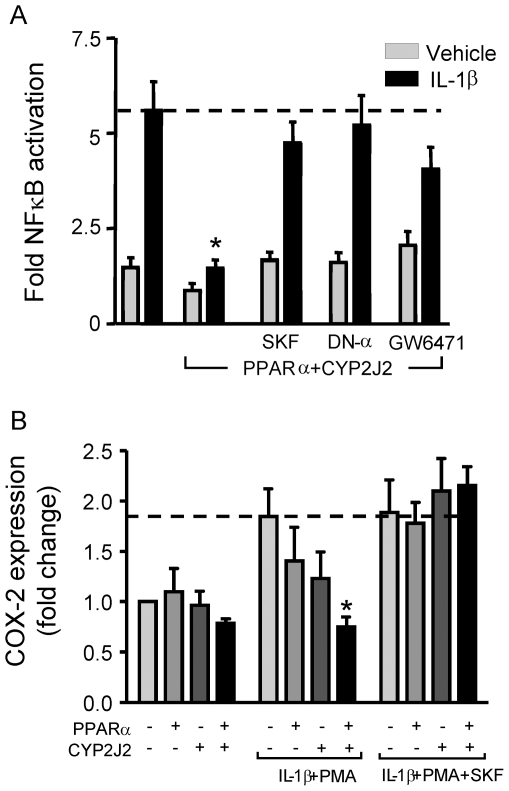
CYP2J2 has anti-inflammatory actions in vitro through the activation of PPARα. (A) CYP2J2 and PPARα co-transfection abolishes IL-1β (10 ng/ml; 18 h) induced NFκB reporter gene activation. HEK293 cells were transfected with a NFκB-luciferase reporter gene and combinations of expression plasmids for CYP2J2 and PPARα, or control empty plasmid (pcDNA3.1). The CYP2J2-PPARα mediated inhibition of IL-1β-induced NFκB activation is reversed by SKF525A (30 µM), co-transfection with dominant negative (DN)-PPARα, or a selective PPARα antagonist GW6471 (3 µM). *denotes p<0.05 between IL-1β and treatments, by one-way ANOVA and Bonferroni's post test. Data represents mean±SEM of n = 9 determinations from 3 separate experiments. (B) CYP2J2 and PPARα co-transfection abolishes IL-1β (10 ng/ml) and PMA (1 nM) induced COX-2 mRNA expression (24 h) determined by semi-quantitative RT-PCR. The abolishment of COX-2 mRNA is reversed when cells are co-incubated with the epoxygenase inhibitor SKF525A (30 µM). *denotes p<0.05 between IL-1β + PMA induced COX-2 expression and the effect of treatments by one-way ANOVA and Bonferroni's post-test. Data represents mean ± SEM of n = 9 determinations from 3 separate experiments.

We have recently shown that CYP2J2 and its products 8,9-EET and 11,12-EET activate PPARα *in vitro* in HEK293 cells, and that CYP2J2 augments PPARα signaling *in vivo* in the heart [10]. Here we show CYP2J2 and PPARα co-transfection abolishes IL-1β induced NFκB reporter gene activation (Figure 1A), or IL-1β and PMA-induced COX-2 mRNA induction (Figure 1B) in HEK293 cells. Transfection with either CYP2J2 or PPARα reduced IL-1β-induced NFκB activation, but both were required to abolish the response (Cont 1.5±0.3; IL-1β 5.6±0.7; IL-1β+CYP2J2 3.3±0.5; IL-1β+PPARα 3.3±0.4; IL-1β+CYP2J2+PPARα 1.5±0.2 fold NFκB activation; CYP2J2 1.3±0.1 or PPARα 1.0±0.1 transfected by themselves did not affect basal NFκB activation). The anti-inflammatory effect of the combination of CYP2J2 activity and PPARα activation could be reversed by either CYP2J2 inhibition with 30 µM SKF525A (Figure 1A and 1B), PPARα inhibition by the PPARα antagonist GW6471 (1 µM) or the presence of co-transfected dominant-negative PPARα (Figure 1A). We previously showed that SKF525A up to concentrations of 30 µM produced a concentration-dependent inhibition of CYP2J2 induced PPAR reporter gene activation [10]. In longer-term experiments however (>24 h), 30 µM SKF525A reduced cell viability (assessed by MTT assay), so 10 µM SKF525A, which had no significant effect on cell viability under any of the conditions tested (data not shown) was used in all the subsequent experiments.

**Figure 2:**
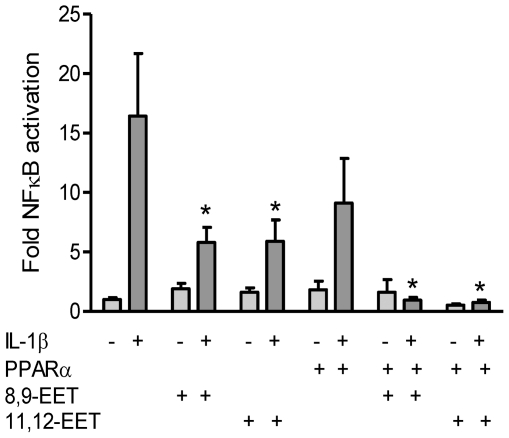
Epoxygenase products inhibit NFκB activation through PPARα-dependent manner and independent pathways. Exogenous EETs inhibit IL-1β-induced NFκB *independently* of PPARα, HEK293 cells were transfected with a NFκB-luciferase reporter gene with either an expression plasmid for PPARα, or control empty plasmid (pcDNA3.1). 8,9-EET and 11,12-EET (1 µM) inhibit IL-1β induced NFκB reporter gene in the presence and absence of co-transfected PPARα. *denotes p<0.05 between IL-1β and treatments, by one-way ANOVA and Bonferroni's post test. Data represents mean ± SEM of n = 9 determinations from 3 separate experiments.

The addition of the known products of CYP2J2, 8,9-EET or 11,12-EET (1 µM) strongly inhibited IL-1β-induced NFκB reporter gene activity in the absence of PPARα (Figure 2). In the presence of transfected PPARα, NFκB activity was abolished using either 8,9-EET or 11,12-EET indicating that EETs may have anti-inflammatory actions both dependent and independent of PPARα (Figure 2).

### Epoxygenase activity in monocytes constitutively limits inflammatory activation in a manner sensitive to PPARα ligands

**Figure 3:**
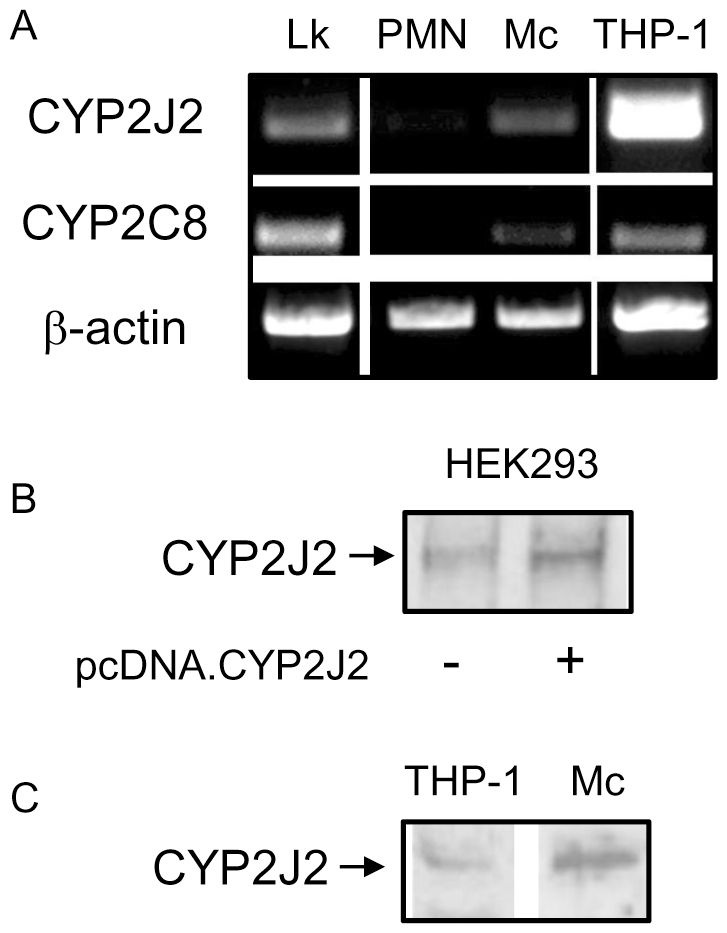
Human monocytes contain epoxygenases CYP2J2 and CYP2C8. (A) CYP2J2 and CYP2C8 mRNA determined by RT-PCR are expressed in human peripheral leukocytes (Lk), monocytes (Mc) and the THP-1 cell line, but not polymorphonuclear cells (PMNs). Confirmation of CYP2J2 protein in human monocytes and THP-1 cells was performed by western blotting. (B) HEK293 cells transfected with empty vector (-) compared to HEK293 cells transfected with expression plasmid for CYP2J2 (+) was used as a positive control. (C) CYP2J2 protein in human THP-1 cells and monocytes (Mc). Figures are representative of n = 3−4 independent experiments.

**Figure 4:**
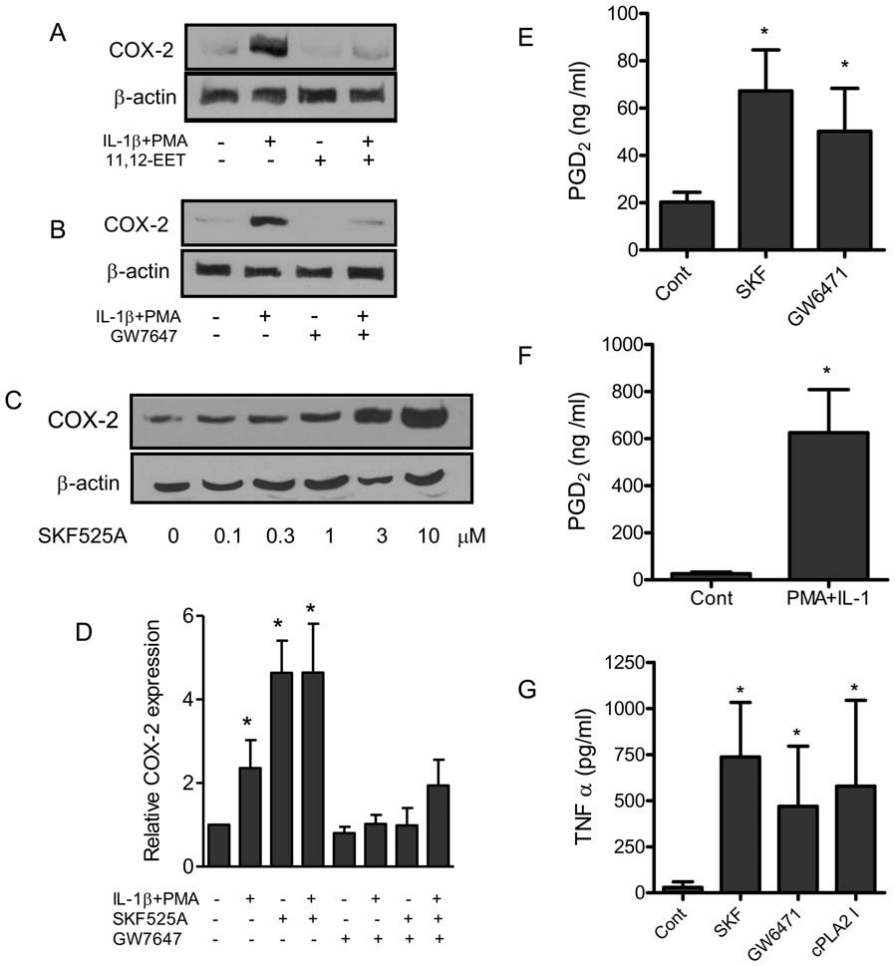
Epoxygenase inhibition induces pro-inflammatory signals in human and murine monocytes in vitro. (A and B) COX-2 protein induced in THP-1 cells by a combination of IL-1β (10 ng/ml) and PMA (1 nM) for 24 h is inhibited by co-incubation with either (A) 11,12-EET (1 µM) or (B) the selective PPARα ligand GW7647 (10 nM). β-actin was measured to ensure equal loading of proteins. Data is representative of n = 3 separate experiments. (C and D) Epoxygenase blockade *alone* is sufficient to induce COX-2 in THP-1 monocytes. (C) SKF525A 0.1–10 µM gives a concentration-dependent induction of COX-2 protein. Data is representative of n = 4 separate experiments. (D) Epoxygenase blockade induces COX-2 in THP-1 cells which is reversed by the selective PPARα agonist GW7647 (10 nM). Compared to the established pro-inflammatory combination of IL-1β and PMA (used at optimal concentrations for COX-2 induction), SKF525A gives a robust induction of COX-2. Data represents mean ± SEM of n = 4 separate experiments. *denotes p<0.05, by one-sample t-test treatments compared to untreated cells. (E) The COX derived prostanoid PGD_2_ is also induced in THP-1 cells by epoxygenase (SKF525A 10 µM) or PPARα blockade (GW6471 10 µM) or (F) a combination of IL-1β (10 ng/ml) and PMA (1 nM) for 24 h. (G) Similarly, TNFα is induced in THP-1 cells by epoxygenase (SKF525A 10 µM), PPARα blockade (GW6471 10 µM) or cPLA_2_ (AACOCF_3_ 30 µM) inhibition over 24 h. *denotes p<0.05 by Wilcoxon signed rank test treatments compared to untreated cells.

CYP2J2 and CYP2C8 (but not CYP2C9) were expressed in human THP-1 monocytes and human peripheral blood monocytes (Figure 3). THP-1 cells produced low levels of the stable epoxygenase products of arachidonic acid: 8,9-EET (8,9-DHET; 2.0±0.3 pg/ml), and linoliec acid, 9,10-epoxy-octadecenoic acid (EPOME) (9,10-dihydroxy-octadecenoic acid, 213.5±111.8 pg/ml) over 24 h. CYP2J2, CYP2C8 or CYP2C9 transcripts were not detected in human PMNs (Figure 3A). Classical activation of monocytes with IL-1β and PMA induced COX-2 protein in a manner sensitive to inhibition by either the epoxygenase product 11,12-EET or the selective PPARα ligand GW7647 [18] (Figure 4A and B). IL-1β alone in both HEK293 or THP-1 cells was insufficient to reproducibly induce COX-2, however the combination of IL-1β with PMA gave a consistent reproducible induction of both COX-2 mRNA and protein. Surprisingly, in the absence of additional stimuli, epoxygenase inhibition using SKF525A alone greatly induced COX-2 protein in THP-1 (Figure 4C and D), which could be reversed by PPARα activation. Similarly, SKF525A induced PGD_2_ release (Figure 4E) and TNFα release (Figure 4G). In addition, the selective PPARα antagonist GW6471 (10 µM) induced PGD_2_ and TNFα release (Figure 4E and 4G), or the cPLA_2_ inhibitor AACOCF_3_ (30 µM; Figure 4G) induced TNFα release similar to SKF525A. The cPLA_2_ inhibitor was not tested for PGD_2_ release as it would directly inhibit PGD_2_ synthesis independent of any effect on EET generation. Although, the level of COX-2 protein induced by epoxygenase inhibition was similar to IL-1β and PMA stimulation, the level of PGD_2_ released were significantly greater with IL-1β and PMA stimulation (Figure 4F). In addition to inflammatory pathways, epoxygenase inhibition also increased the uptake of Dil-labeled acetylated-LDL in a manner sensitive to inhibition with GW7647 (Figure S1). In THP-1 cells, no uptake was observed until the monocytes had been activated with 1 nM PMA for 24 h, before treatment with SKF525A.

### Epoxygenase activity differentially regulates alternatively activated monocytes and macrophages

**Figure 5:**
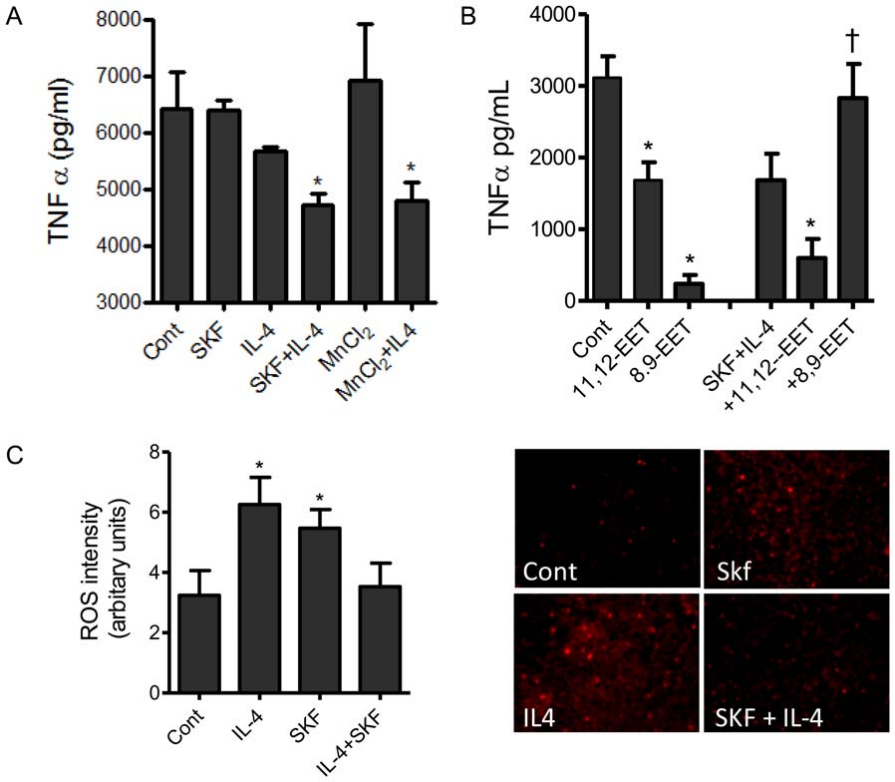
Epoxygenase inhibition in alternatively activated monocytes inhibits TNFα release: a role for superoxide generation. (A) shows the acute release of TNFα from THP-1 cells over 7 h with vehicle treatment (Cont), or treated with SKF525A (10 µM), IL-4 (20 ng/ml), the superoxide dismutase, MnCl_2_ (10 µM) or a combination of IL-4 and SKF525A or IL-4 and MnCL_2_. (B) shows inhibition of basal TNFα release from THP-1 cells treated with 11,12-EET or 8,9-EET (1 µM) in the presence of absence of SKF (10 µM) with IL-4 (20 ng/ml). Data represents mean ± SEM of n = 3 experiments. * indicates EET difference from control, while † indicates EET difference from IL-4 with SKF525A. (C) shows the production of superoxide (left figure; arbitrary units), or representative florescent micrographs (right panels), from THP-1 cells treated for 7 h with vehicle treatment (Cont), SKF525A (10 µM), IL-4 (20 ng/ml), or a combination of IL-4 and SKF525A. The superoxide sensor, dihydroethidium (10 µM) was added for the last 30 min and staining intensity analyzed using a Nikon TE2000 inverted florescent microscope connected to a SPOT-RT digital camera. Data represents mean ± SEM of n = 3−4 experiments. *denotes p<0.05, by one-way ANOVA and Bonferroni's post-test compared to control cells.

THP-1 monocytes treated with the alternative activator of monocytes IL-4 (20 ng/ml) exhibited a small reduction in TNFα release over 7 h (Figure 5A). In cells treated with IL-4 and the epoxygenase inhibitor SKF525A (10 µM), TNFα release was further reduced (Figure 5), suggesting that epoxygenases are immuno-modulators depending on the underlying activation state of the monocyte. Similar results were found for COX-2 (and TNFα) mRNA levels (Figure S2A and B). Under basal cell culture conditions 11,12-EET and 8,9-EET strongly inhibited TNFα release. In contrast, in the presence of IL-4 and SKF525A, although 11,12-EET retained its inhibitory activity, 8,9-EET elevated TNFα levels (Figure 5B), suggesting a shift in the cells ability to sense this anti-inflammatory EET. A change in the sensitivity to an anti-inflammatory EET may therefore help to explain this pro-inflammatory action of IL-4 under these conditions.

Moreover, since epoxygenases can activate pro-inflammatory pathways via superoxide generation [9], we monitored superoxide generation and compared SKF525A responses to that of the superoxide dismutase compound MnCl_2_ [19]. MnCl_2_ (10 µM) identical to epoxygenase inhibition decreased TNFα release in the presence but not absence of IL-4 (Figure 5A), showing that superoxide anion generation from epoxygenases can account for the reduction in TNFα release under conditions of alternative activation. Superoxide generation was increased in both IL-4 (20 ng/ml) and SKF525 (10 µM) treated cells, the combination of IL-4 with SKF525 however showed no significant increase of superoxide generation over control treated cells (Figure 5C).

**Figure 6:**
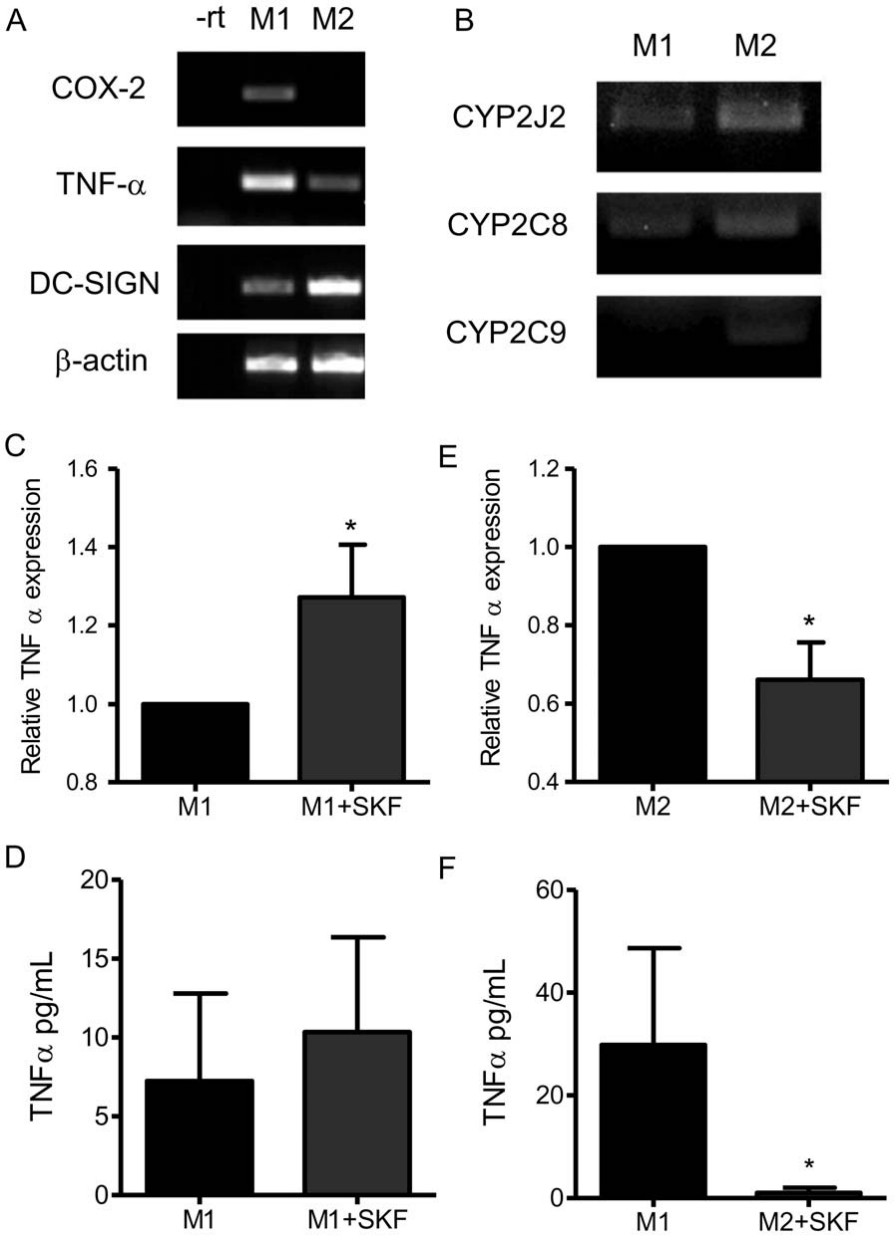
Epoxygenase inhibition differentially regulates the release of TNFα from M1 and M2 macrophages. (A) Differentiation of human peripheral blood mononuclear cells for 7d with either LPS (1 ng/ml) and IFNγ (20 ng/ml; M1) or IL-4 (20 ng/ml; M2) induces a characteristic pattern of M1 (high COX-2, high TNFα, low DC-SIGN) and M2 (low COX-2, low TNFα and high DC-SIGN) macrophage differentiation. Markers were measured by RT-PCR compared to β-actin. (B) CYP2J2 and CYP2C8 mRNA are expressed in M1 and M2 macrophages. In addition CYP2C9 is expressed in (2 out of 4) macrophages. Blots are representative of n = 4 independent experiments. (C-F) TNFα mRNA (top) and protein (lower panels) is differentially regulated in M1 (C and D): primary M1 macrophages (C) and M1-THP-1-macrophages (D), and M2 (E and F): primary M2 macrophages (E), and M2-THP-1 (F) macrophages treated with SKF525A (10 µM). Monocytes were placed in differentiation medium containing vehicle or SKF525A for 7d. M1 and M2-THP-1 cells were identically treated with M1 or M2 differentiation media, however in these experiments, SKF525A (10 µM) was given to fully differentiated cells at the end of the experiment on day 7 for a further 7 h. The relative TNFα mRNA compared to β-actin levels was measured by RT-PCR. TNFα release was determined by ELISA. *denotes p<0.05, by one-sample t-test compared to untreated cells.

We differentiated human peripheral blood monocytes in to M1 and M2 macrophages with LPS (1 ng/ml) and IFNγ (20 ng/ml), or with IL-4 respectively, in the presence or absence of SKF525A (10 µM) for 7 days (Figure 6). M1 macrophages were characterized by high transcript levels of COX-2 and TNFα, and low levels of DC-SIGN (Figure 6A), in contrast M2 macrophages showed no COX-2 expression, lower levels of TNFα, but high levels of the M2 marker DC-SIGN (Figure 6A). M1 and M2 macrophages expressed CYP2J2 and CYP2C8 mRNA. In addition, CYP2C9 mRNAs were also detected in two cultures of M2 macrophages (Figure 6B). In experiments where SKF525A was included during the differentiation process, identical to our findings in monocytes, M1 macrophages had increased TNFα mRNA (Figure 6C and D), while M2 macrophages had significantly reduced TNFα mRNA levels (Figure 6E and 6F). TNFα release by ELISA could not be accurately measured in M1 and M2 macrophages due to low and variable cell numbers. We therefore measured TNFα release in THP-1 cells treated with M1 and M2 differentiation medium for 7 days. In these experiments, SKF525A treatment was only given for a 7 h period at the end of the experiment and not throughout. SKF525A did not affect the release of TNFα in M1-THP-1 cells (Figure 6D), but greatly reduced the release of TNFα by M2-THP-1 cells (Figure 6F). The absolute levels of TNFα release were less in M1 compared to M2-THP-1 cells due to a large reduction in cell viability with the M1 differentiation conditions. SKF525A at this 7 h time point did not however effect cell viability in either M1 or M2 differentiated cells.

## Discussion

The roles of epoxygenase pathways in monocytes and macrophages remain poorly understood. Here we show endogenous epoxygenases provide a critical endogenous break on monocyte/macrophage cell inflammatory pathway activation under resting conditions and when activated to differentiate to the M1 phenotype. We choose to use the non-specific epoxygenase inhibitor SKF525A as there are at least 2 epoxygenases present and we wanted to ensure we had a complete inhibition of these epoxygenases. PPARα, CYP2J2 and EETs have all been shown independently to have anti-inflammatory properties, at least in part by inhibition of NFκB. Consistent with our previous findings that co-transfected CYP2J2 inhibited NFκB through providing ligands for PPARα [20], here we show that the NFκB downstream inflammatory targets COX-2 and TNFα are similarly regulated. Epoxygenase products, which we previously showed capable of activating PPARα [10], inhibited NFκB activation in the absence of transfected PPARα. Although the inhibition of NFκB by these EETs was complete when PPARα was present, since our HEK293 cells do not contain any significant level of basal PPAR activity [10], these results suggest that anti-inflammatory EETs can act through both PPAR-dependent and PPAR-independent pathways. Recently epoxygenases and their products were shown to regulate inflammation at the level of the endothelial cell neutrophil interactions [13]. Although PPARγ has also been suggested as an anti-inflammatory target for endothelial epoxygenases [21], [22], the reduction of pro-inflammatory cytokines induced by epoxygenases were reversed by the putative EET antagonist 14,15-EEZE [13]. It must be noted however that 14,15-EEZE does not have pure antagonist properties [23], and has yet to be tested on the regulation of PPAR responses. One additional potential anti-inflammatory mechanism for EETs is via cAMP generation. This does not appear to be the case here as SKF525A had no affect on cAMP levels in THP-1 cells, at least after 7 h of treatment (Fig S2C).

Monocytes and macrophages contain PPARα [24] and here we show they express CYP2J2 and CYP2C8, and release low levels of epoxygenase products of arachidonic acid, and linoleic acid. Interestingly, the stable levels of EETs release were much lower than the levels of the linoleic acid product 9,10-EPOME. As yet we don't understand the importance of these alternative epoxygenase fatty acid products in monocytes, but in our hands 9,10-EPOME does not have anti-inflammatory properties similar to 11,12-EET as it does not inhibit basal TNFα release from THP-1 cells (Fig S2D). PPARα knockout monocyte/macrophages exist in a highly inflammatory state producing large amounts of thrombospondin-1 and inhibiting tumor growth [25]. Inhibition of monocyte epoxygenases using SKF525A strongly induced the NFκB target genes COX-2 and TNFα, consistent with our *in vitro* co-transfection studies. Indeed, inhibition of either epoxygenases, or PPARα using the selective antagonist GW6471 induced prostanoid release, or along with a cPLA_2_ inhibitor AACOCF_3_, induced TNFα release to a similar extent, indicating that inhibiting any of the components of a proposed pathway: cPLA_2_ - arachidonic acid - epoxygenase - EET - PPARα, results in a similar monocyte activation. The increased expression of COX-2 was sensitive to inhibition by 11,12-EET (the EET we previously described with the greatest potency to activate PPARα) or the highly selective PPARα ligand GW7647 [18]. These results show that epoxygenases supply a continuous anti-inflammatory tone in monocytes at least in part by producing PPARα ligands.

It has been recently reported that CYP2J2 was absent in human leukocytes, but present at high levels in malignant hematological (lymphocytic) cell lines and in leukemia cells from peripheral blood and bone marrow in patients with malignant hematologic diseases [26]. Although we found CYP2J2 and CYP2C8 mRNA in human total leukocytes, lymphocyte and monocyte fractions (but not PMNs), the levels of mRNA were consistently lower than those found in our THP-1 cells (a human acute monocytic leukemia cell line). The high levels of CYP2J2 in these cancers may help to explain some of the poor immunological function of these cells, and may point to the presence of a high degree of continuous immuno-suppression through this pathway.

In contrast, when alternatively activated by IL-4, concurrent epoxygenase inhibition instead of elevating TNFα suppressed TNFα release and TNFα mRNA expression in THP-1 monocytes or M2 macrophages respectively. Similar results were found with COX-2 in THP-1 monocytes: however, there was no basal level of COX-2 in M2 macrophages to modulate. These results show that under alternative activation, epoxygenases have a pro-inflammatory activity. Interestingly, IL-4 with SKF525A changed the actions of the epoxygenase product 8,9-EET when given exogenously. Under basal conditions 8,9-EET inhibited TNFα release, while in the presence of IL-4 and SKF525A it elevated TNFα levels. These intriguing results point to a new pro-inflammatory EET pathway in monocytes sensitized by IL-4 which is specific to 8,9-EET, as 11,12-EET was unaffected. The best characterized pro-inflammatory activity of epoxygenases is however, through the production superoxide anion [9]. In our experiments TNFα release was suppressed by epoxygenase inhibition with SKF525A, to the same extent as with removal of superoxide by the superoxide dismutase MnCl_2_ [19]. Whether this is accompanied by a change in anti-inflammatory EET signaling is not clear. Intriguingly, the M2 phenotype was accompanied by an induction of CYP2C9 mRNA, the epoxygenase best characterized as a superoxide generator. SKF525A or IL-4 induced superoxide generation in THP-1 monocytes, however the combination of SKF525A and IL-4 showed no increase in superoxide anion production. These results suggest that IL-4 induces a switch in the profile of epoxygenase activity to a pro-inflammatory superoxide generating system. Although IL-4 is generally considered anti-inflammatory, IL-4 has previously reported to increase as well as decrease both superoxide anion generation [27], [28] and NFκB activation [29], [30] in monocytes depending on the underlying stimulation.

Our results show an epoxygenase–PPARα axis provides a critical endogenous break on monocyte/macrophage during classical activation. This inflammatory breaking signal therefore represents a novel regulatory mechanism in monocyte/macrophages that may regulate disease progression [31], [32]. During alternative activation endogenous epoxygenases limited the anti-inflammatory activity of IL-4 via superoxide generation, and potentially by changing the monocyte/macrophage sensitivity to EETs, highlighting a novel additional immunoregulatory role for epoxygenases during Th2 mediated immune responses.

## Methods

### Ethics Statement

Ethics to take blood samples from healthy volunteers were approved by the St Thomas's Hospital Research Ethics Committee (07/Q0702/24), and conducted according to the Declaration of Helsinki. All volunteers gave written informed consent prior to entering the study.

### Materials

NFκB-luc reporter plasmid was from Clontech (Takara Bio Europe/Clontech, Saint-Germain-en-Laye, France). Rabbit polyclonal anti-CYP2J2 was raised as previously described [33]. pCMXmPPARα and dominant negative DN-(h6/29) hPPARα were gifts from Dr Ruth Roberts (AstraZeneca; Maccelsfield, U.K.). EETs and the PGD_2_ ELISA were from Cayman Chemical Company (Cambridge Bioscience, Cambridge, UK). SKF525A was from Biomol (Affiniti Research Products, Exeter, UK). NovaFECTOR was from VennNova (Pompano Beach, FL, USA). Dihydroethidium and Dil labelled acetylated-(ac)-LDL conjugate was from InVitrogen (Paisley, Renfrewshire, UK). cAMP HTRF assay was from Cisbio (Codolet, France). ECL reagents, hyperfilm were from GE Healthcare (Little Chalfont, Buckinghamshire, UK). Cytokines were from R&D Systems (Abingdon, Oxfordshire, UK). Unless stated, all other reagents were from Sigma-Aldrich (Poole, Dorset, UK).

### Cell and tissue culture

HEK293 (ATCC; LGC Standards, Middlesex, UK) were cultured in DMEM, and THP-1 were cultured in RPMI supplemented with antibiotic/antimycotic mix, and 10% FBS; 37°C; 5% CO2; 95% air. Primary monocytes were isolated from peripheral blood of human volunteers as previously described [34]. Four million cells per well were left to adhere to the culture plastic for 1.5 hr at which point non-adherent cells were discarded. M1 and M2 differentiation was achieved by stimulation of the adhered monocytes or PMA treated THP-1 cells with 1 ng/mL LPS (*E. coli* serotype 026:B6) and 20 ng/mL IFNγ for the M1 phenotype and 20 ng/ml IL-4 for the M2 phenotype for four days. The concentrations of the cytokines for M1 and M2 differentiations were selected from two publications [35], [36].

Cell morphology, dihydroethidium staining (10 µM for 30 min at the end of the experiment) [37] and Dil-acLDL uptake (5 µg/ml for 24–72 h) was analyzed using a Nikon TE2000 inverted florescent microscope connected to a SPOT-RT digital camera. The figures shown were created from unmodified images from 2–6 random low magnification fields (x200), analyzed for mean florescent intensity using ImageJ image analysis software (http://rsb.info.nih.gov/ij/). The photo-images shown for the purposes of clarity have been modified in an identical manner in Powerpoint (contrast and brightness). Luciferase assays were performed as previously described [38].

### RT-PCR

Cell and tissue samples analyzed by RT-PCR were lysed and RNA isolated using Trizol^TM^ (Invitrogen). Human β-actin [39] and COX-2 [40] were as previously described. Human TNF-alpha gene forward primer 5′-CAG AGG GCC TGT ACC TCA TC-3′ and reverse primer 5′-GGA AGA CCC CTC CCA GAT AG-3′ producing a 218-bp fragment. DC-SIGN, forward primer: 5′-AGT GGG TGA GCT TCC AGA GA-3′ and reverse primer 5′-CTA AAT TCC GCG CAG TCT TC-3′ producing a 405-bp fragment. CYP2J2, forward primer 5′-GCC CGG GAG TCC ATG CCC TA-3′ and reverse primer 5′-AAC AGC GCA GAG GCG GTG AC-3′ producing a 435-bp fragment. CYP2C8, forward primer 5′-CGG TGT GCC CCA TGC AGT GA-3′and reverse primer AGA TCG GCA GCC AGA TGG GC-3′ producing a 411-bp fragment. CYP2C9, forward primer ATT GAC CTT CTC CCC ACC AGC-3′ and reverse primer GCA AAT CCA TTG ACA ACT GGA GT producing a 355-bp fragment. Amplification was performed in a Techgene Techne TC-312 thermal cycler programmed for an initial denaturation of 5 min at 94°C, followed by 25–35 cycles of 1 min at 94°C, 1 min at 57–60°C and 2 min at 72°C, and a final extension of 7 min at 72°C. 12 µl of the PCR reaction was subjected to electrophoresis on 1.5% agarose gel and the products visualized by ethidium bromide staining. The gel image was captured with a Gel doc 1000 BIORAD digital camera.

### Western Blotting, immunoassays, and EET measurements

CYP2J2, COX-2, PPARα, and β-actin protein levels were measured as previously described [33], [38]. CYP2J2 antibody cross-reacts with CYP2J subfamily enzymes but not other isoforms [33]. The intensity of the bands was digitalized, analyzed and quantified with the Image J Software Package (NIH). TNFα, PGD_2_ ELISAs and cAMP HTRF assays were performed according to manufacturer's instructions. LC/MS/MS analysis of epoxygenase products in culture supernatants were measured as previously described [41].

## Supporting Information

Figure S1 **Epoxygenase inhibition increase macrophage lipid uptake.** SKF525A treatment (10 µM; 72 h) increases the uptake of DiL-acetylated LDL which is inhibited if co-treated with the selective PPARα ligand GW7647 (100 nM). Cells were initially pre-treated with PMA (5 nM) for 24 h. Left panels show representative micrographs (x400) of ac-LDL uptake, figure shows densometric analysis (Image J) of ac-LDL uptake represented as mean ± SEM of n = 4 experiments.
(PDF)

Figure S2 **(A & B) shows COX-2 mRNA (C) and TNFα mRNA (D) relative to β-actin in THP-1 cells treated with vehicle (Cont), or treated with SKF525A (10 µM), IL-4 (20 ng/ml or IL-4 with SKF525A for 7 h.** Data represents mean ± SEM of n = 4 experiments. * indicates p<0.05; one way ANOVA compared to control. (C) Intracellular cAMP levels (pg/ml) are unchanged in THP-1 cells treated with vehicle control (Cont) or SKF525A (10 µM) for 7 h. (D) Shows the comparison of inhibition of basal TNFα release from THP-1 cells treated with 1 µM of 11,12-EET or 9,10-EPOME. Data represents mean ± SEM of n = 3 experiments.
(PDF)
