# Supplementary material for: Endogenous Epoxygenases Are Modulators of Monocyte/Macrophage Activity
Source: PLoS One. 2011 Oct 19;6(10):e26591. doi: 10.1371/journal.pone.0026591 (PMC3197524; doi:10.1371/journal.pone.0026591)
Supplement: Figure S2 — (A & B) shows COX-2 mRNA (C) and TNFα mRNA (D) relative to β-actin in THP-1 cells treated with vehicle (Cont), or treated with SKF525A (10 µM), IL-4 (20 ng/ml or IL-4 with SKF525A for 7 h. Data represents mean ± SEM of n = 4 experiments. * indicates p<0.05; one way ANOVA compared to control. (C) Intracellular cAMP levels (pg/ml) are unchanged in THP-1 cells treated with vehicle control (Cont) or SKF525A (10 µM) for 7 h. (D) Shows the comparison of inhibition of basal TNFα release from THP-1 cells treated with 1 µM of 11,12-EET or 9,10-EPOME. Data represents mean ± SEM of n = 3 experiments. (PDF) [file pone.0026591.s002.pdf]

Supplemental Figure 2

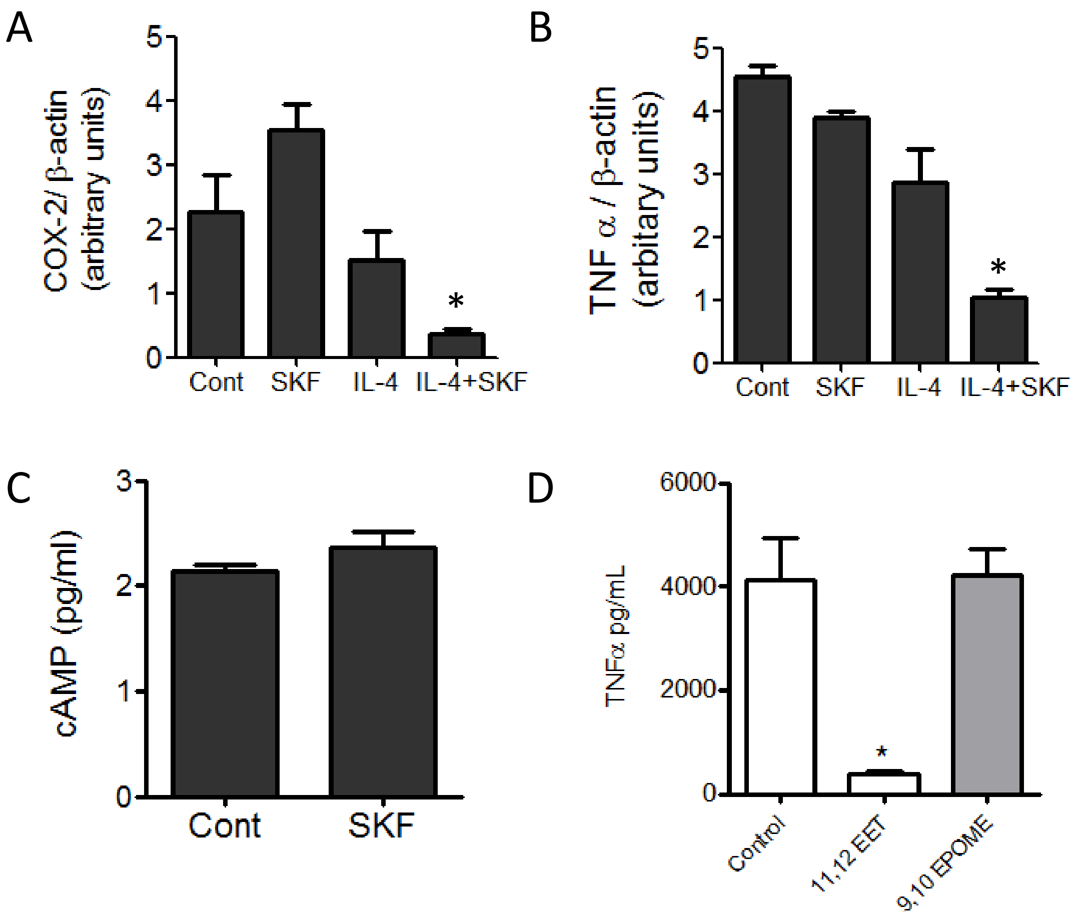

Supplemental Figure S2: (A & B) shows COX-2 mRNA (C) and TNFα mRNA (D) relative to β-actin in THP-1 cells treated with vehicle (Cont), or treated with SKF525A (10μM), IL-4 (20ng/ml or IL-4 with SKF525A for 7h. Data represents mean ± SEM of n=4 experiments. * indicates $p<0.05$; one way ANOVA compared to control. **(C)** Intracellular cAMP levels (pg/ml) are unchanged in THP-1 cells treated with vehicle control (Cont) or SKF525A (10μM) for 7h. **(D)** Shows the comparison of inhibition of basal TNFα release from THP-1 cells treated with 1μM of 11,12-EET or 9,10-EPOME. Data represents mean ± SEM of n=3 experiments.
